# Pericoronary Adipose Tissue Radiomics from Coronary Computed Tomography Angiography Identifies Vulnerable Plaques

**DOI:** 10.3390/bioengineering10030360

**Published:** 2023-03-14

**Authors:** Justin N. Kim, Lia Gomez-Perez, Vladislav N. Zimin, Mohamed H. E. Makhlouf, Sadeer Al-Kindi, David L. Wilson, Juhwan Lee

**Affiliations:** 1 Department of Biomedical Engineering, Case Western Reserve University, Cleveland, OH 44106, USA; 2 Department of Biomedical Engineering, The Ohio State University, Columbus, OH 43210, USA; 3 Cardiovascular Imaging Core Laboratory, Harrington Heart and Vascular Institute, University Hospitals Cleveland Medical Center, Cleveland, OH 44106, USA; 4 Department of Radiology, Case Western Reserve University, Cleveland, OH 44106, USA

**Keywords:** pericoronary adipose tissue, microchannel, microvessel, thin-cap fibroatheroma, optical coherence tomography, coronary computed tomography angiography, machine learning

## Abstract

Pericoronary adipose tissue (PCAT) features on Computed Tomography (CT) have been shown to reflect local inflammation and increased cardiovascular risk. Our goal was to determine whether PCAT radiomics extracted from coronary CT angiography (CCTA) images are associated with intravascular optical coherence tomography (IVOCT)-identified vulnerable-plaque characteristics (e.g., microchannels (MC) and thin-cap fibroatheroma (TCFA)). The CCTA and IVOCT images of 30 lesions from 25 patients were registered. The vessels with vulnerable plaques were identified from the registered IVOCT images. The PCAT-radiomics features were extracted from the CCTA images for the lesion region of interest (PCAT-LOI) and the entire vessel (PCAT-Vessel). We extracted 1356 radiomic features, including intensity (first-order), shape, and texture features. The features were reduced using standard approaches (e.g., high feature correlation). Using stratified three-fold cross-validation with 1000 repeats, we determined the ability of PCAT-radiomics features from CCTA to predict IVOCT vulnerable-plaque characteristics. In the identification of TCFA lesions, the PCAT-LOI and PCAT-Vessel radiomics models performed comparably (Area Under the Curve (AUC) ± standard deviation 0.78 ± 0.13, 0.77 ± 0.14). For the identification of MC lesions, the PCAT-Vessel radiomics model (0.89 ± 0.09) was moderately better associated than the PCAT-LOI model (0.83 ± 0.12). In addition, both the PCAT-LOI and the PCAT-Vessel radiomics model identified coronary vessels thought to be highly vulnerable to a similar standard (i.e., both TCFA and MC; 0.88 ± 0.10, 0.91 ± 0.09). The most favorable radiomic features tended to be those describing the texture and size of the PCAT. The application of PCAT radiomics can identify coronary vessels with TCFA or MC, consistent with IVOCT. Furthermore, the use of CCTA radiomics may improve risk stratification by noninvasively detecting vulnerable-plaque characteristics that are only visible with IVOCT.

## 1. Introduction

The development of atherosclerotic plaques can lead to acute coronary syndrome, with plaque rupture being the primary cause. One of the key precursors of plaque rupture is thin-cap fibroatheroma (TCFA) [1,2], and the presence of microchannels (MC) in plaques also indicates increased plaque vulnerability and intraplaque hemorrhage [3,4]. The early identification of these vulnerable plaque characteristics is critical for effective risk assessment and treatment planning.

Intravascular optical coherence tomography (IVOCT) is currently the only imaging modality with the ability to detect TCFAs and MCs with its high axial resolution of 12–18 µm. Coronary computed tomography angiography (CCTA) is widely used as a first-line assessment tool to evaluate coronary artery disease (CAD) [5,6,7,8]. It can assess the plaque burden and identify high-risk-plaque characteristics (HRP), such as low-attenuation plaque, napkin ring sign, positive remodeling, and spotty plaque calcification, which are associated with future acute coronary syndrome (ACS) [9,10]. 

Pericoronary adipose tissue (PCAT) has been shown to play a critical role in the pathophysiology of atherosclerosis by releasing adipokines and cytokines, which increase inflammation and contribute to the progression of the disease [11,12,13]. Previous studies have reported increased mean PCAT attenuation around culprit lesions in patients with ACS compared to nonculprit lesions [14], as well as increased mean PCAT attenuation at the RCA and the lesion in stable CAD patients with HRP compared to those without HRP [15].

Radiomics has recently emerged as a promising tool in CCTA for the quantification and analysis of various tissue features [16]. Several studies have demonstrated the feasibility of radiomics analysis in the assessment of PCAT in CCTA images, showing that PCAT radiomics can distinguish myocardial ischemia and improve cardiac-risk prediction [17,18,19]. However, the association between PCAT radiomics and vulnerable-plaque characteristics, such as TCFA and MC, as assessed by IVOCT, has not yet been explored. 

In this study, we aimed to investigate the relationship between PCAT-radiomics features from CCTA images and vulnerable-plaque characteristics, as assessed by IVOCT. Our work is novel in its focus on the investigation of this association, which has not been previously explored in the literature. While building on our previous studies of IVOCT-image analysis [20,21,22] and the Optical Coherence TOmography PlaqUe and Stent (OCTOPUS) analysis software [23,24,25,26,27,28], our study differs in its use of radiomics to analyze CCTA-derived PCAT-radiomics features.

We used OCTOPUS [29] to register IVOCT and CCTA images and analyze features at both the lesion and whole-vessel levels. A machine-learning approach was used to determine whether PCAT-radiomics features from CCTA could be used to predict the presence of plaque-vulnerability characteristics (TCFAs and MCs), as identified by IVOCT. Through this investigation, our study may offer insights into the underlying biological mechanisms of atherosclerosis by exploring the relationship between PCAT radiomics and vulnerable-plaque characteristics.

## 2. Materials and Methods

### 2.1. Study Population

This study retrospectively identified 25 patients from University Hospitals (Cleveland, OH, USA) who underwent both CCTA and IVOCT procedures based on medical indications. Exclusion criteria were as follows: (1) history of myocardial infarction, (2) previous coronary stent implantation, and (3) poor quality of CCTA or IVOCT images. The study was approved by the Institutional Review Board of University Hospitals (Cleveland, OH, USA) and carried out in accordance with the Declaration of Helsinki’s principles. Written informed consent was waived for this retrospective study. 

### 2.2. IVOCT Imaging

The IVOCT images were obtained from a C7XR frequency-domain OCT Imaging System (Abbott Vascular, Santa Clara, CA, USA) after an injection of nitroglycerin (100–200 g). The IVOCT was performed with Dragonfly OPTIS 2.7 F 135 cm. Blood clearance was achieved by non-diluted iodine contrast using ISOVUE-370 (iopamidol injection, 370 mg iodine/mL; Bracco Diagnostics Inc., Princeton, NJ, USA). The optical probe employed automated pullback at a rate of 36 mm/s using survey mode (375 frames, 75 mm), a frame rate of 180 frames/s, and axial resolution of 20 μm. The IVOCT images were deidentified and analyzed at the Cardiovascular Phenomics Core at University Hospitals. Expert cardiologists with more than 9 years of experience assessed the quality of each pullback for inclusion in the analysis.

### 2.3. The Use of IVOCT Processing to Extract Vulnerability Characteristics

We employed the OCTOPUS software, which was developed in previous studies [20,21,22,23,24,25,26,27,28,29], to identify TCFA and MC from IVOCT images. This software utilizes deep-learning algorithms to detect and classify these vulnerable-plaque characteristics efficiently and accurately [26,27,28]. We then applied binary labeling to each coronary vessel in the presence of TCFA and MC. The TCFA was defined as a plaque whose thinnest fibrous cap measured less than 65 µm and had a TCFA angle greater than 90°. Meanwhile, MC was defined as a non-signal, tubuloluminal structure that was not connected with the vessel lumen and was recognized on three or more consecutive cross-sectional IVOCT images [4]. 

### 2.4. The CCTA Acquisition

All CCTA images were acquired on a Brilliance ICT 256 scanner (Philips Healthcare, Cleveland, OH, USA), in accordance with institutional clinical protocols. BMI-specific scanner settings were used (BMI ≤ 30 kg/m^2^: 100 kV/300 mAs; BMI > 30 kg/m^2^: 120 kV/450 mAs for prospective gating; and BMI ≤ 30 kg/m^2^: 100 kV/800 mAs, BMI > 30 kg/m^2^: 120 kV/800 mAs, for retrospective gating), with craniocaudal scan direction. In cases with irregular rhythm, we used retrospective gating without tube modulation. A total of 80 mL iodinated contrast dye (ISOVUE 370) was injected via 18-gauge angiocath 20 or 22 Diffusics needle (Nexiva™, BD, NJ, USA), preferably at 5–6 mL/s, followed by a 70-milliliter saline flush, divided into a 20-milliliter test flush at a rate of 6 mL/s prior to the scan and a 50-milliliter bolus chase after contrast injection. The bolus was tracked in the ascending aorta at the level of the carina with a threshold of 100 Hounsfield Unit (HU) for optimal imaging. Hyperemia was achieved by sublingual nitroglycerin 5 min prior to scan, and a targeted heart rate of 60 or less was achieved using metoprolol 100 mg PO and intravenous metoprolol in increments of 5 mg up to 20 mg if systolic pressure was >100 mmHg. 

To facilitate analysis of coronary vessels on CCTA images, we utilized a multiplanar reformatted approach using an Aquarius workstation (version 4.4.11-13; TeraRecon, Foster City, CA, USA) to produce images of straightened vessels. From the resulting straightened vessels, axial slices of coronary vessels were obtained and stored as DICOM files. These files were then transmitted via a secure connection for further segmentation and analysis of PCAT. The use of multiplanar reformatted images and axial slices allowed a more precise and standardized analysis of the coronary vessels. 

### 2.5. The PCAT Segmentation

The segmentation of PCAT in CCTA images was carried out with the following steps. First, the vessel walls were segmented using an Aquarius workstation, and the resulting segmentations were reviewed and corrected by expert-cardiologist readers to ensure accuracy. The PCAT was defined as the regions of interest consisting of voxels within a radial distance of the outer coronary wall equal to the vessel diameter, with a CT attenuation value between −190 HU and −30 HU [11,14,18,30]. We used our in-house Python programs to segment the PCAT candidate regions by importing the previously segmented vessel walls and applying the criteria to select the appropriate PCAT regions. To extract the PCAT mask, the CT attenuation values were then thresholded between −190 HU and −30 HU. The PCAT was segmented for each coronary vessel and exported as binary masks for subsequent radiomic-feature extraction. On average, the PCAT segmentation time for each vessel was approximately 50 s, making this process efficient and reliable for subsequent analyses.

### 2.6. Radiomic Analysis

The extracted radiomic features were grouped into three categories—shape, intensity, and texture. Shape-based features describe the shape and geometric properties calculated independently of the gray-level intensity distribution. Intensity (first-order) features depend on the distribution of HU values (e.g., mean, standard deviation, entropy, skewness, kurtosis, etc.) without considering the spatial distribution. Texture features calculate the statistical interrelationship between neighboring voxels [31]. For example, gray-level co-occurrence matrices (GLCM) describe the frequency of co-occurrences of a HU pixel value pair; gray-level dependence matrix (GLDM) quantifies a number of connected voxels within a distance that is dependent on the center voxel [32]; gray-level run length matrix (GLRLM) quantifies the frequency of consecutive occurrences of the same voxel value [33]; gray-level size-zone matrix (GLSZM) quantifies homogeneity and variation characteristics by measuring the number of voxels with the same value [31,33]; and neighboring gray-tone difference matrix (NGTDM) describes the central and neighboring pixels [33]. 

The voxels of each vessel were discretized into three specified bin sizes of 8, 16, and 32 bins, with equal HU ranges. To increase the number of features based on the distribution of the extracted radiomic features, the minimum, maximum, mean, and standard deviation were calculated. Of the 1356 radiomic features extracted, 252 were shape features, 204 were intensity features, and 900 were texture features. On average, the time taken to extract radiomic features and eliminate highly correlated features for each vessel was approximately 9 min. The open-source PyRadiomics package [34] in Python was utilized for the calculation of radiomic features, in accordance with Image Biomarker Standardization Initiative (IBSI) guidelines [35].

### 2.7. The PCAT-Radiomics Feature Extraction

To enable spatial correlative analysis of data, we manually registered IVOCT pullbacks to corresponding vessels in CCTA images by identifying landmark characteristics of the coronary artery, such as bifurcations or large calcifications. Two ranges of CCTA frames were selected for PCAT-radiomic-feature extraction: (1) the CCTA-IVOCT registered plaque lesion of interest, PCAT-LOI; and (2) the entire set of CCTA frames of the coronary artery, PCAT-Vessel. The PCAT-Vessel range started from the ostium for the LAD and LCX, while the first 10 mm from the ostium were excluded for the RCA. The end point of the PCAT-Vessel range was the end of the coronary arteries. The PCAT was then segmented from the selected CCTA frame ranges and radiomic features were extracted.

### 2.8. Association of PCAT Radiomics with IVOCT Vulnerable-Plaque Characteristics

To examine the associations between CCTA-derived PCAT radiomics and IVOCT vulnerable-plaque characteristics, the following analyses were conducted. Three binary classes were established to categorize the vessels with TCFA, MC, and both TCFA and MC (IVOCT-TCFA, IVOCT-MC, and IVOCT-TCFA-MC, respectively). From the initial 1356 radiomic features generated from both PCAT-LOI and PCAT-Vessel models, we removed the features that showed strong correlations (|r| > 0.95). The remaining 293 features for PCAT-LOI and 341 features for PCAT-Vessel were used for further analysis. 

Univariate analysis was conducted to evaluate the diagnostic performance of each individual features. For each binary class, we applied univariate logistic regression to each radiomic feature obtained from PCAT-LOI and PCAT-Vessel models and used stratified three-fold cross-validation with 1000 repetitions. We calculated the average receiver operating characteristic (ROC) curve and the area under the ROC curve (AUC) to assess the diagnostic performance of each feature. The mean AUC of each feature in identifying the presence of TCFA and MC was visualized using a Manhattan plot.

Multivariate analysis was performed to evaluate the overall diagnostic performance of the radiomics model based on feature selection. From the univariate logistic regression, 15 features with the highest mean AUC in each of seven classes of features (shape, first-order, and five subclasses of texture features) were selected. The minimum redundancy maximum relevance (mRMR) method is a feature selection technique used in radiomics research that selects a subset of relevant and non-redundant features [36]. It can help to reduce the dimensionality of high-dimensional radiomic datasets, improve the accuracy and generalizability of radiomics models, and mitigate issues of multicollinearity and overfitting, all of which can compromise the reliability and robustness of the model. We used the mRMR algorithm to select a subset of 50 features out of 105 that were highly correlated with the output class and had the least mutual information. The feature-selection process was then repeated to refine the model to a maximum of 10 features. This allowed us to identify the most relevant and non-redundant features for predicting the presence of IVOCT-TCFA, IVOCT-MC, and IVOCT-TCFA-MC.

The final radiomics model was refined to retain 10 features at most, and the optimal set of features was chosen recursively by removing the features of least importance, as evaluated by the coefficient. The feature-selection process was repeated to obtain the PCAT-LOI and PCAT-Vessel radiomics models for the prediction of IVOCT-TCFA, IVOCT-MC, and IVOCT-TCFA-MC. 

The features included in the final radiomics model are shown Table S1 in Supplementary Materials. Due to the limited sample size of the vessel dataset, the overall diagnostic performance may not have been generalizable. To address this issue and reduce overfitting bias, we utilized stratified three-fold cross-validation with 1000 repetitions to obtain a robust estimation of the models’ performance on new datasets. The mean area under the receiver operating characteristic curve (AUC) and its standard deviation (SD) were calculated to assess the diagnostic performances of the models. The feature selection and statistical analysis were carried out on Python using the Pymrmr (https://pypi.org/project/pymrmr/) and Scikit-learn (https://pypi.org/project/scikit-learn/) open-source library [37]. 

## 3. Results

### 3.1. Patient and Lesion Characteristics

Table 1 summarizes the clinical characteristics of the study population. Overall, 30 lesions from 25 patients were analyzed. The mean patient age was 63 ± 11 years, and six patients had prior CABG. Most of the patients were male (75%) and had diabetes mellitus (87%) and dyslipidemia (96%). There were no statistically significant associations between the clinical characteristics and the presence of TCFA or MC. Most of the lesions were in the left anterior descending artery (LAD; 76.7%). The median (interquartile range (IQR)) time between the CCTA and IVOCT procedures was 11 (3–24) days. Of the 30 lesions, IVOCT-TCFA was present in fourteen, IVOCT-MC in twelve, and IVOCT-TCFA-MC in six lesions.

**Figure 1:**
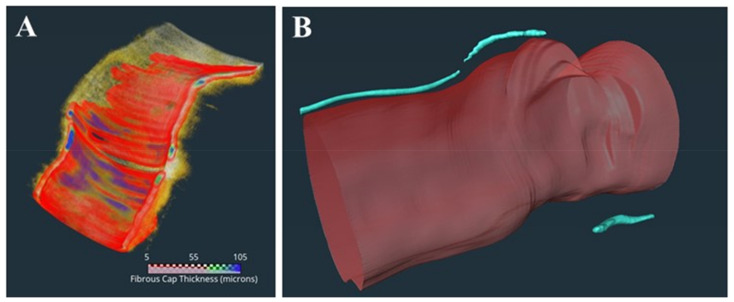
Three-dimensional (3D) visualization of IVOCT coronary-artery segments with (**A**) TCFA and (**B**) MC. A heatmap of fibrous-cap thickness is overlayed in (**A**), showing the TCFA region. As described in the text, TCFA (red) was defined as a plaque with a fibrous cap < 65 µm and TCFA angle > 90° for each frame. The MC was detected as described in the text. In this instance, there were three microvessels (blue) in this plaque. The microvessels’ segments were 7.4 mm in length and their diameters were approximately 10.4 µm. Multiple radiomic features captured the extent of TCFA and microvessel presence in plaques.

**Figure 2:**
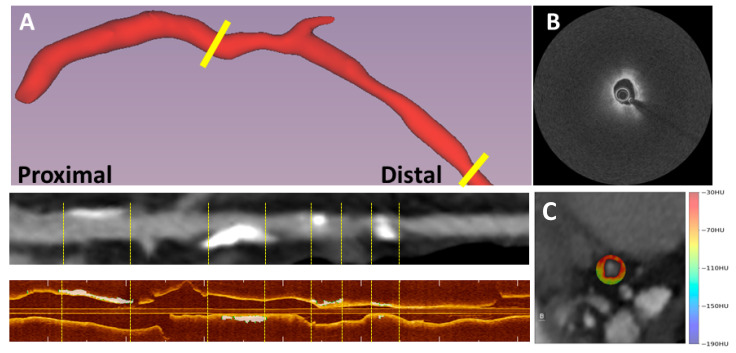
Registration of CCTA and IVOCT images. In panel (**A**), 3D CCTA coronary vessel (**top**), the straightened CCTA (**middle**), and the IVOCT coronary vessel (**bottom**). Registration was performed using our developed software, OCTOPUS. Note that the white calcified plaques in the straightened CCTA view correspond to matching calcifications in IVOCT, demonstrating good registration. In panel (**B**), an IVOCT axial frame of a non-calcified lesion is shown. Panel (**C**) shows a registered CCTA axial frame overlayed with HU colormap segmentation of PCAT.

Figure 1A,B, respectively, show the rendering of the OCTOPUS-identified fibrous-cap thickness and microchannels along a coronary artery. Figure 2A shows the outcome of the registration of the CCTA and IVOCT coronary-vessel images by OCTOPUS. The calcified plaque, shown in white, is well aligned between the imaging modalities. Figure 2B shows the CCTA axial slice of a coronary vessel, overlayed with PCAT segmentation of the defined HU range.

### 3.2. Univariate Anlaysis of PCAT Radiomics to Identify IVOCT-TCFA and IVOCT-MC 

**Figure 3:**
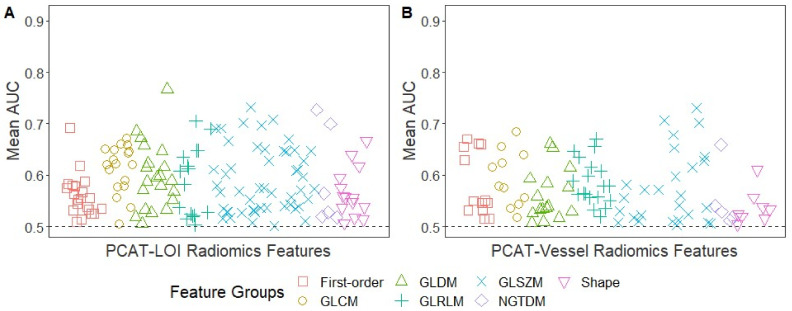
Univariate CCTA-feature analysis for predicting IVOCT-TCFA. Manhattan plot of PCAT-LOI (**A**) and PCAT-Vessel (**B**) of the mean AUCs for the identification of coronary vessels with IVOCT-TCFA. The number of radiomic features with AUC > 0.5 for identification of coronary vessels with IVOCT-TCFA was 147/293 (50.2%) for PCAT-LOI and 99/341 (29.0%) for PCAT-Vessel. Of the 147 PCAT-LOI-radiomics features with AUC > 0.5, 14 (9.5%) were shape features, 21 (14.3%) were first-order statistics, and 112 (76.2%) were texture-based features (GLCM: 18, GLDM: 21, GLRLM: 16, GLSZM: 51, NGTDM: 6). Of the 99 PCAT-Vessel-radiomics features with AUC > 0.5, eight (8.1%) were shape features, thirteen (13.1%) were first-order statistics, and seventy-eight (78.8%) were texture-based features (GLCM: 11, GLDM: 16, GLRLM: 19, GLSZM: 27, NGTDM: 5).

After removing the highly correlated PCAT-radiomics features, we analyzed the association of the remaining features with the vulnerable-plaque characteristics on the IVOCT. Of the 293 PCAT-LOI-radiomics features (Figure 3A), six (2.0%) had mean a AUC between 0.70 and 0.79, 50 (17.1%) had values between 0.60 and 0.69, and 91 (31.1%) had values between 0.50 and 0.59 in the identification of coronary vessels with IVOCT-TCFA. Of the 341 PCAT-Vessel-radiomics features (Figure 3B), three (3.0%) had mean AUC between 0.70 and 0.79, twenty-six (26.3%) had values between 0.60 and 0.69, and seventy (70.7%) had values between 0.50 and 0.59 in the identification of coronary vessels with IVOCT-TCFA. The radiomic features with the highest AUC was the minimum dependence variance for the PCAT-LOI (AUC = 0.77, GLDM, discretized at 16 equally sized bins), and the mean size-zone non-uniformity normalized for the PCAT-Vessel (AUC = 0.73, GLSZM, discretized at 32 bins).

**Figure 4:**
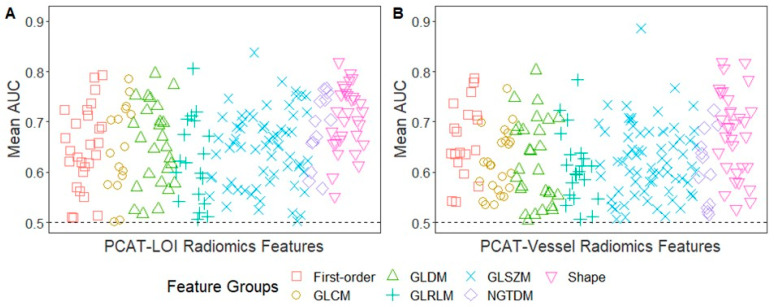
Univariate CCTA-radiomics feature analysis for predicting IVOCT-MC. Manhattan plot of PCAT-LOI (**A**) and PCAT-Vessel (**B**) of the mean AUCs for the identification of coronary vessels with IVOCT-MC. The number of radiomic features with AUC > 0.5 for identification of coronary vessels with IVOCT-MC was 195/293 (66.6%) for PCAT-LOI and 203/341 (59.5%) for PCAT-Vessel. Of 195 radiomic features with AUC > 0.5 from the PCAT-LOI, 31 (15.9%) were shape features, 27 (13.9%) were first-order statistics, and 137 (70.3%) were texture-based features (GLCM: 16, GLDM: 26, GLRLM: 19, GLSZM: 63, NGTDM: 13). Of 203 radiomic features from the PCAT-Vessel, 32 (15.8%) were shape features, 19 (9.4%) were first-order statistics, and 152 (74.9%) were texture-based features (GLCM: 24, GLDM: 28, GLRLM: 21, GLSZM: 67, NGTDM: 12).

Of the two hundred and ninety-three PCAT-LOI-radiomics features (Figure 4A), three (1.0%) had a mean AUC between 0.80 and 0.89, sixty-four (21.8%) had a value between 0.70 and 0.79, seventy-five (25.6%) were between 0.60 and 0.69, and fifty-three (18.1%) were between 0.50 and 0.59 in the identification of coronary vessels with IVOCT-MC. Of the 341 PCAT-Vessel-radiomics features (Figure 4B), five (1.5%) had a mean AUC between 0.80 and 0.89, thirty-seven (10.9%) had a value between 0.70 and 0.79, eighty-two (24.0%) were between 0.60 and 0.69, and 79 (23.2%) were between 0.50 and 0.59 in the identification of coronary vessels with IVOCT-MC. The radiomic features with the highest mean AUC were the maximum small-area high gray-level emphasis (GLSZM, discretized at eight equally sized bins) for both the PCAT-LOI (AUC = 0.84) and the PCAT-Vessel (AUC = 0.89).

### 3.3. Multivariate Analysis of PCAT Radiomics to Identify IVOCT-TCFA and IVOCT-MC

**Figure 5:**
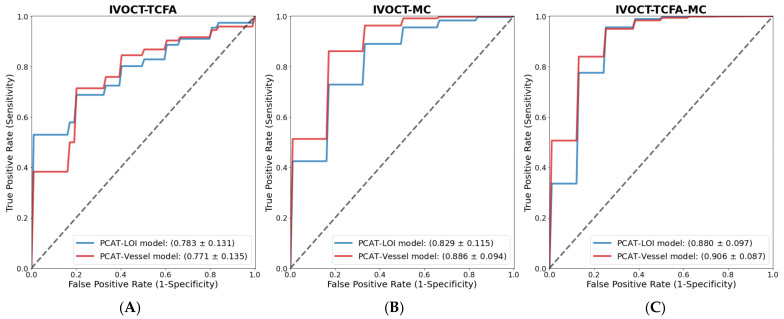
Multivariate analysis of CCTA PCAT-radiomic features associated with IVOCT vulnerable-plaque characteristics on IVOCT. Diagnostic performance obtained from the three-fold cross-validation with 1000 repeats for the identification of (**A**) IVOCT-TCFA, (**B**) IVOCT-MC, and (**C**) IVOCT-TCFA-MC (IVOCT-Vulnerable). The ROC curves of PCAT-radiomics models for PCAT-LOI and PCAT-Vessel are plotted, and the mean AUC and SD are reported.

We built radiomic models after a series of feature-selection procedures. For the identification of coronary vessels with IVOCT-TCFA, the ROC curves of the PCAT-LOI and PCAT-Vessel radiomics models were examined, and they are plotted in Figure 5A. The PCAT-LOI and PCAT-Vessel models retained three and five radiomic features, respectively. The mean AUC of the PCAT-LOI radiomics model was 0.783 (SD = 0.131) and that of the PCAT-Vessel radiomics model was 0.771 (SD = 0.135). See Table S1 in Supplementary Materials for the final radiomic features used in each model. 

For the identification of coronary vessels with IVOCT-MC, the ROC curves of the PCAT-LOI and PCAT-Vessel radiomics models are plotted in Figure 5B. Three radiomic features were retained in the PCAT-LOI and PCAT-Vessel models (Supplementary Table S1). The mean AUC of the PCAT-LOI radiomics model was 0.829 (SD = 0.115) and that of the PCAT-Vessel radiomics model was 0.886 (SD = 0.094). 

### 3.4. Radiomic Features for Identifying Vulnerable Vessels 

To assess the ability of PCAT radiomics to identify the most vulnerable plaques with both TCFA and MC (IVOCT-TCFA-MC), a multivariate analysis was conducted using the same feature-selection and model-building methods. The PCAT-LOI and PCAT-Vessel models retained three and eight radiomic features, respectively, with no overlapping features in the final models. 

Figure 5C shows the ROC curves of the radiomics models in the identification of vulnerable coronary vessels, IVOCT-TCFA-MC. The mean AUC of the PCAT-LOI radiomics model was 0.880 (SD = 0.097) and that of the PCAT-Vessel radiomics model was 0.906 (SD = 0.087). See Supplementary Table S1 for the final radiomic features used in each model.

## 4. Discussion

Our findings demonstrate that the PCAT-radiomics features on CCTA images are associated with microscopic findings of plaque vulnerability, such as thin-cap fibroatheroma (TCFA) and microchannels (MC), in IVOCT. To the best of our knowledge, this is the first study to use PCAT radiomics to identify coronary vessels with TCFA and MC. These results have important implications for the utility of CCTA in the early identification and risk assessment of vulnerable plaques. 

Our study may elucidate the potential role of PCAT in the development of atherosclerosis and its relationship with the occurrence of TCFA and MC. Microchannels are newly formed microvessels that originate from the vasa vasorum and have been linked to increased lipid influx and macrophage infiltration into coronary plaques [38]. Moreover, the presence of MCs in IVOCT images has been shown to be predictive of plaque vulnerability. Specifically, a higher frequency of MCs on IVOCT is associated with a greater frequency of TCFA and positive remodeling [4]. While these vulnerable-plaque characteristics are not captured on CCTA due to the limited spatial resolution, CCTA provides non-invasive-imaging data, providing an opportunity to analyze indiscernible spatial patterns through radiomics. 

Various studies attempted to identify IVOCT-TCFA using CCTA. Previous studies [39,40,41] attempted to utilize CCTA high-risk plaque characteristics but produced conflicting findings. This may have been due to the interobserver variability in the visual assessment of high-risk-plaque features [42]. Radiomics, which offers quantitative metrics, has been applied to CCTA images to identify IVOCT-TCFA. Kolossváry et al. demonstrated that CCTA radiomics identified TCFA on IVUS, IVOCT, and NaF^18^-PET with good-to-excellent accuracy [43]. Chen et al. reported that a coronary-plaque radiomics model outperformed conventional high-risk-plaque features in identifying IVOCT-TCFA [44]. Although the limited spatial resolution of CCTA images does not allow the visualization of microscopic vulnerable-plaque characteristics, such as TCFA and MC, radiomics may quantify unique distribution patterns of HU-containing vulnerable plaques. While previous studies demonstrated the feasibility of coronary-plaque radiomics analysis in the identification of vulnerable-plaque features, they often overlooked the potential role of PCAT in atherosclerosis and its possible association with TCFA and MC. Our study explicitly focuses on the investigation of the relationship between PCAT-radiomics features and the presence of vulnerable-plaque characteristics, which may offer novel insights into the underlying biological mechanisms of atherosclerosis.

In our study, we found several PCAT-radiomics features that were predictive of vulnerable-plaque characteristics on IVOCT through univariate analyses of their association with TCFA and MC. Specifically, the PCAT-LOI feature class had a greater percentage of features with AUC > 0.5 for the association with IVOCT-TCFA compared to the PCAT-Vessel features (50.2% vs. 29.0%, respectively). This suggests a potential relationship between PCAT and the presence of TCFA, which is consistent with the outside-in theory of atherosclerosis. This theory posits that inflamed adipocytes in PCAT contribute to the development of atherosclerosis via the production of adipocytokines. Presumably, inflammation leads to morphological changes in PCAT (e.g., increased water retention in adipocytes and changes in vascularity), which can be captured by the radiomic features.

Our PCAT-radiomics models demonstrated good accuracy in identifying vulnerable plaque characteristics (TCFA and MC). Interestingly, the PCAT-LOI feature class tended to have higher AUCs when identifying IVOCT-TCFA compared to the PCAT-Vessel feature class. The PCAT-Vessel slightly outperformed the PCAT-LOI in identifying IVOCT-MC. These results align with the morphological occurrence of vulnerable-plaque characteristics, as TCFA occurs in discrete lesions, while MC tends to occur along the lengths of coronary vessels. 

Although our study has limitations, including a retrospective design with a relatively small number of datasets obtained from a single center, we employed rigorous statistical methods to account for this limited sample size. Stratified three-fold cross-validation with 1000 repeats was used to calculate the AUCs and ensure the robustness of our models. We also combined all vessel types, which were independently assessed. Future studies with larger datasets and multi-center cohorts are warranted to further validate our results.

Overall, our findings suggest that PCAT-radiomics features extracted from CCTA may provide useful information for identifying vulnerable-plaque characteristics. All the PCAT-radiomics models successfully identified IVOCT-TCFA, IVOCT-MC, and IVOCT-TCFA-MC on the CCTA images. The quantitative radiomics analysis of CCTA images may also enable the identification of other microstructures in coronary plaques, such as cholesterol crystals and macrophage infiltration. The integration of CCTA-radiomics-imaging biomarkers can further improve cardiac-risk assessment and treatment planning. 

## 5. Conclusions

Our results indicate that non-invasive CCTA-derived PCAT radiomics can identify vessels with IVOCT vulnerable-plaque characteristics, such as thin-cap fibroatheroma and microchannels. 

## Figures and Tables

**Table 1:** Clinical variables of the study population. Values are mean ± standard deviation (SD) for continuous variables, and *n* (%) for categorical variables. The FHx of CAD, family history of coronary artery disease; CABG, coronary-artery-bypass graft; eGFR, estimated glomerular filtration rate; WBC, white blood cell; LAD, left anterior descending artery; LCx, left circumflex artery, RCA, right coronary artery. * Two missing records.

Clinical Characteristics	*n* = 25
Age, years	63 ± 11
Male	19 (76.0%)
Body Mass Index, kg/m^2^	28.5 ± 4.7
Cardiovascular Risk Factors	
Hypertension	11 (44.0%)
Diabetes	20 (87.0% *)
Chronic Kidney Disease	11 (44.0%)
Family History of CAD	14 (56.0%)
Prior CABG	11 (44.0%)
Dyslipidemia	24 (96.0%)
Blood Parameters	
Creatinine eGFR	1.5 ± 1.7
WBC count, ×10^9^/*l*	15.1 ± 35.4
Hemoglobin, g/dL	12.9 ± 1.9
Hematocrit, %	39.4 ± 5.0
Platelet count, ×10^9^/*l*	285.5 ± 85.3
HDL-c, mg/dL	42.1 ± 7.6
LDL-c, mg/dL	127.0 ± 44.9
Triglycerides, mg/dL	175.3 ± 88.3
Total-c, mg/dL	200.3 ± 50.8
Lesion Characteristics	*n* = 30
Lesion Location	
LAD	23 (76.7%)
LCx	4 (13.3%)
RCA	3 (10.0%)

## Data Availability

The data presented in this study are available on request from the corresponding author.

## Supplementary Materials

The following supporting information can be downloaded at: https://www.mdpi.com/article/10.3390/bioengineering10030360/s1. Table S1: List of features included in the final models of identification of IVOCT-TCFA, IVOCT-MC, and IVOCT-TCFA-MC.
