# Supplementary material for: Pericoronary Adipose Tissue Radiomics from Coronary Computed Tomography Angiography Identifies Vulnerable Plaques
Source: Bioengineering (Basel). 2023 Mar 14;10(3):360. doi: 10.3390/bioengineering10030360 (PMC10045206; doi:10.3390/bioengineering10030360)
Supplement: Supplementary file 1 [file bioengineering-10-00360-s001.zip › bioengineering-2235613-supplementary.pdf]

bioengineering

MDPI

Table S1. List of features included in the final models of identification of IVOCT-TCFA, IVOCT-MC, and IVOCT-TCFA-MC, respectively. Favorable radiomics features tended to be those describing texture and size of PCAT.

IVOCT-TCFA	
**PCAT-LOI model**	**PCAT-Vessel model**
max_glszm_SizeZoneNonUniformity_bin32	max_shape_MinorAxisLength_bin8
std_gldm_LargeDependenceEmphasis_bin8	min_ngtdm_Coarseness_bin8
min_glszm_GrayLevelNonUniformityNormalized_bin16	max_firstorder_Range_bin8
	max_glrlm_RunLengthNonUniformity_bin16
	mean_glszm_SizeZoneNonUniformityNormalized_bin32

IVOCT-MC	
**PCAT-LOI model**	**PCAT-Vessel model**
mean_ngtdm_Busyness_bin32	min_glszm_SizeZoneNonUniformityNormalized_bin16
mean_shape_Sphericity_bin8	max_shape_Maximum2DDiameterSlice_bin8
std_shape_Maximum2DDiameterSlice_bin8	std_firstorder_Mean_bin8

IVOCT-TCFA-MC	
**PCAT-LOI model**	**PCAT-Vessel model**
std_gldm_DependenceNonUniformityNormalized_bin32	max_shape_Maximum2DDiameterSlice_bin8
max_glszm_SmallAreaHighGrayLevelEmphasis_bin16	std_glrlm_LongRunHighGrayLevelEmphasis_bin8
mean_glszm_SizeZoneNonUniformityNormalized_bin8	min_gldm_LowGrayLevelEmphasis_bin8
	max_shape_MinorAxisLength_bin8
	mean_ngtdm_Busyness_bin32
	max_glcm_ClusterShade_bin8
	max_glszm_SmallAreaHighGrayLevelEmphasis_bin8
	std_shape_Maximum2DDiameterRow_bin8
